# New Developments in PCR-Based Diagnostics for Bacterial Pathogens Causing Gastrointestinal Infections—A Narrative Mini-Review on Challenges in the Tropics

**DOI:** 10.3390/tropicalmed6020096

**Published:** 2021-06-02

**Authors:** Ulrike Loderstädt, Ralf Matthias Hagen, Andreas Hahn, Hagen Frickmann

**Affiliations:** 1 Institute for Infection Control and Infectious Diseases, University Medical Center Göttingen, 37075 Göttingen, Germany; ulrike.loderstaedt1@med.uni-goettingen.de; 2 Department of Microbiology and Hospital Hygiene, Bundeswehr Central Hospital Koblenz, Andernacher Str. 100, 56070 Koblenz, Germany; ralfmatthiashagen@bundeswehr.org; 3 Institute for Medical Microbiology, Virology and Hygiene, University Medicine Rostock, 18057 Rostock, Germany; Hahn.andreas@me.com; 4 Department of Microbiology and Hospital Hygiene, Bundeswehr Hospital Hamburg, 20359 Hamburg, Germany

**Keywords:** PCR, gastroenteritis, tropics, pre-analytics, interpretation, validation, diagnostics, infection, colonization, nucleic acid extraction

## Abstract

The application of modern PCR approaches for the diagnosis of bacterial gastrointestinal pathogens is on the rise due to their rapidly available results combined with high sensitivity. While multiple studies describe the ongoing implementation of this technique for routine diagnostic purposes in laboratories in Western industrialized countries, reports on successful and also sustainable respective approaches in resource-poor tropical settings are still scarce. In order to shed light on potential reasons for this marked discrepancy, this narrative review summarizes identified challenges for the application of diagnostic PCR targeting bacterial gastrointestinal pathogens from stool samples in the tropics. The identified and discussed issues comprise the lack of generally accepted definitions for (1) minimum standards regarding sample acquisition, storage and transport time for diagnostic PCR analyses in the tropics, (2) nucleic acid extraction standards allowing an optimum detection of all types of pathogens which may be responsible for gastroenteritis in the tropics, (3) validation standards to ensure comparable quality of applied diagnostic assays, and (4) cut-offs for a reliable discrimination of infection and mere colonization in areas where semi-immunity due to repeated exposition associated with poor hygiene conditions has to be expected. Further implementation research is needed to solve those issues.

## 1. Introduction

As of April 2021, academic interest in PCR-based diagnosis of gastroenteritis has been documented by the more than 7000 articles on the NCBI (National Center for Biotechnology Information) PubMed database (https://pubmed.ncbi.nlm.nih.gov/, last accessed on 3 May 2021). Among those articles, only about 2% are focused on the specific situation in the tropics. This obvious neglect reflects the situation of scarce availability of this technology in the laboratories of resource-poor tropical countries; however, it does not necessarily indicate a lacking local need.

On the contrary, as early as in 1994 and thus only seven years after the invention of PCR [1], a Swiss study reported on the successful application of diagnostic PCR targeting shigellae as well as enteroinvasive and enterotoxigenic *Escherichia coli* in returnees from the tropics [2]. PCR-based identification of the diarrheagenic *E. coli* [2] was a considerable achievement for diagnostic laboratories, because colony morphology of diarrheagenic and non-diarrheagenic *E. coli* is usually indistinguishable on agar plates.

More than 25 years have gone by since those very first approaches. In some laboratories in resource-rich Western industrialized countries, PCR targeting bacterial causes of gastroenteritis has even been established as a new screening standard with subsequent culture and resistance testing only in case of positive PCR results [3]. Some authors from such well-equipped laboratories have even argued that cost reductions due to the implementation of PCR panels might be achieved [4]. This, of course, only applies assuming that PCR technology is readily available and costs for laboratory personnel are the limiting factor. Such prerequisites, however, are not always granted, so the authors’ conclusions [4] might even be considered as cynical in some resource-poor tropical regions.

Accordingly, PCR targeting gastrointestinal pathogens acquired in the tropics has widely remained a domain of returnee assessments [5,6] or study settings [7]. For returnee screening purposes, even elaborated interpretation strategies for the case of multiple positive results recorded due to the application of multiplex PCR panels have been introduced [8].

Within the tropics itself, experience with PCR-based routine diagnosis of gastrointestinal pathogens is scarce, making the establishing of evidence-based guidelines challenging. As known from the data within the Geosentinel database on diseases acquired from international travel [9,10] as well as from experience with travelers in the tropics by our own group and others [5,11,12], infectious causes of gastroenteritis are bacteria, eukaryotic parasites and viruses in declining order in resource-poor tropical countries while viruses are most frequently etiologically relevant in Western industrialized countries followed by bacteria and parasites. In particular, diarrheagenic *E. coli* and *Shigella* spp., *Campylobacter* spp., and *Salmonella enterica* dominate in the tropics as shown in previous studies [5,7,11,13,14,15,16,17,18,19]. Cold-affine *Yersinia* spp. and spore-forming *Clostridioides difficile* are considerably less frequently detected in tropical settings [7,13]; *Tropheryma whipplei* persists in tropical climates but usually just as a harmless enteric colonizer [20,21], and *Vibrio* spp. are usually associated with acute outbreak events [22].

In line with those reports, the focus of the review presented here is on chances and challenges of PCR targeting causative agents of bacterial gastroenteritis with particular emphasis on tropical conditions.

## 2. Methods

The search terms “PCR”, “gastroenteritis”, “bacteria”, and “tropics” were applied in various combinations with the database NCBI PubMed. Selected articles as well as the authors’ own practical experience were chosen to write a narrative review on diagnostic PCR targeting gastroenteric pathogens in the tropics. In particular, challenges of the diagnostic processes, beginning from pre-analytic considerations and ending with result interpretation issues, were within the focus of this work.

## 3. Pre-Analytics–on Storage and Transport Conditions

As stated above, diagnostic PCR targeting gastroenteritis-associated pathogens has most frequently been applied in study settings in the tropics so far. Thereby, it is often unfeasible to provide fully equipped PCR laboratories at all remote study sites, so samples are usually transferred to a central diagnostic infrastructure. Accordingly, a delay between sample acquisition and further processing of the samples for the diagnostic process is usually unavoidable.

Although well-funded studies may provide options for cooled or even frozen sample storage and transport, such optimum conditions are not always granted [23]. While successful preservation and stabilization of DNA and RNA in purified nucleic acid eluates has been described for 30 days irrespective of storage temperature, the quality of non-extracted nucleic acids tends to gradually decline over time in spite of stabilizing matrix effects within the sample [24]. Therefore, various strategies have been discussed to circumvent the potentially arising problem of target DNA degradation due to prolonged sample storage.

Preservation of nucleic acids on Whatman cards, which are known to stabilize bacterial DNA for at least three years at ambient temperature [25], has been introduced as a promising approach. In a recent study with spiked stool samples, comparable positivity rates for bacterial agents causing gastroenteritis were observed after nucleic acid extraction from Whatman cards and directly from the spiked stool samples [26]. Promising results were also shown for DNA of protozoan parasites in stool after storage on Whatman cards under laboratory conditions [27].

However, alternative strategies had been assessed as well. Stabilization of pathogen nucleic acids on swabs with proprietary drying systems was successfully applied for the molecular diagnosis of gastrointestinal pathogens with acceptable diagnostic accuracy [28,29].

In a recently performed “real-life” observation in tropical Tanzania [30], bacterial DNA in stool samples both on Whatman cards and on nucleic acid-stabilizing swabs remained stable for several months of storage at ambient temperature. Positivity rates of multiplex PCR were comparable after immediate nucleic acid extraction from stool and after delayed nucleic acid extraction from Whatman cards and from swabs. However, imperfect agreement as defined by Cohen’s kappa [31] was observed [30], a finding which is not easy to interpret. Differing sensitivity due to parallelism of variable free nucleic acid degradation and variable nucleic acid release from decaying cells is one potential explanation. Another likely explanation is release of cross-reacting DNA of non-target organisms after prolonged storage. Accordingly, a residual uncertainty regarding diagnostic accuracy remains [30].

In spite of innovative approaches as mentioned above, standardization approaches in terms of defining evidence-based minimum requirements for pre-analytic conditions prior to PCR for gastroenteric pathogens in stool samples in the tropics are widely missing. Large multicentric, preferably multinational, studies would be desirable to identify widely accepted minimum standards based on sufficiently large datasets.

## 4. Nucleic Acid Extraction–Challenges for a “One-Size-Fits-All” Solution

After arrival of samples in the diagnostic laboratory, nucleic acid extraction is usually the first analytic step. Stool samples are not the easiest specimens for PCR, because they include various inhibitory components [32]. However, three decades of experience with several studies on the optimization of nucleic acid extraction from stool [32,33,34,35] have guided the way to quite reliable nucleic acid extraction protocols from stool samples. Thereby, interestingly, automated nucleic acid extraction did not always outcompete traditional manual column-based nucleic acid extraction [35]. Accordingly, it may remain a question of operational practicability, whether more man power can be provided for laborious manual extraction procedures [35] or whether a slightly higher inhibition rate as controlled by inhibition control PCR [36] can be accepted.

While the complex and inter-individually variable composition of stool samples implies the likely presence of multiple different inhibitory components, some of them such as, e.g., bile salts, hemoglobin degradation products and complex polysaccharides, have been more thoroughly assessed [37,38,39,40,41]. Strategies to overcome inhibition comprise sample dilution or purification applying separation columns, which may in turn result in decreased sensitivity for samples in which the target DNA amounts are close to the detection threshold [42,43,44,45,46,47]. Other approaches include the use of PCR facilitating components such as, e.g., the polyamine compound spermidine [37,48,49] to reduce negative effects of PCR inhibitors. Modern commercial nucleic acid extraction assays for stool samples usually contain preparation steps for the reduction of the inhibition problem.

In case of diarrhea due to enteroinvasive bacteria like *Campylobacter* spp., *Salmonella* spp., or *Shigella* spp./enteroinvasive *E. coli*, not only stool samples but also incubated blood culture material may be considered for PCR-based analysis for diagnostic purposes or in tropical study settings [50]. In scientific literature, it is a rarely addressed topic that nucleic acid extraction from incubated blood culture materials with various standard procedures including column-based and automatic approaches leads to sample inhibition, making the resulting eluates unsuitable for PCR analysis [50,51,52]. It is possible to circumvent this problem by centrifugation-based protocols [50,51,52]. However, those approaches are pretty time-consuming and laborious, so they are hardly suitable for routine diagnostic use in times of MALDI-TOF-MS (matrix-assisted laser-desorption ionization time-of-flight mass spectrometry) application for species identification from incubated blood culture materials [53,54,55].

In addition, sample inhibition is not the only challenge for nucleic acid extraction. When stool samples from the tropics are assessed, the diagnostic focus is rarely on bacterial pathogens alone, but parasitic infectious agents are frequently also targeted [56,57,58]. In order to release nucleic acids from strong-shelled eggs or cuticle cells of helminthic parasites, harsh nucleic acid extraction methods, i.e., freeze-thawing at the very least [59] but also more robust, bead beating-based extraction schemes [60,61] have been suggested, although their superiority in terms of sensitivity has not been continuously demonstrated [62]. For protozoan parasites, similarly harsh nucleic acid extraction procedures have been proposed as well [63,64].

In the diagnostic routine situation, it is if not unfeasible, at least inconvenient to have different nucleic acid extraction schemes for different types of pathogens. In any way, such a requirement will not facilitate the implementation of PCR technology at resource-limited sites, so compromises regarding sensitivity are frequently made. If harsh bead beating-based nucleic acid extraction as recommended for molecular helminth screenings is applied for PCRs targeting bacterial pathogens, sensitivity is comparable regarding the proportions of positive results compared to column-based standard nucleic acid extraction [62]. However, agreement as indicated by Cohen’s kappa [31] leaves room for improvement [62] and it is not always clear whether only sensitivity but also specificity of the PCR approaches is affected by the different extraction approaches [62] in a similar way as discussed above for pre-analytic conditions.

Accordingly, there is neither standardization of defined minimum requirements regarding the quality of nucleic acid extraction from stool samples if a broad spectrum of tropical pathogens is desired, nor agreement on which compromises regarding diagnostic sensitivity are considered acceptable for a “one-size-fits-all”-solution for nucleic acid extraction from stool prior to target-specific PCR. PCR from stool samples after nucleic acid extraction with commercially available standard nucleic acid extraction kits in line with the manufacturers’ recommendations has been shown to be similarly sensitive as culture for the diagnosis of bacterial pathogens [3] and even more sensitive than microscopy for the diagnosis of enteric protozoa [65] in stool samples. Uncertainty, however, remains regarding the reliability of PCR for the diagnosis of enteric helminths in stool samples in comparison to microscopy [62]. Future studies addressing the definition of optimized nucleic acid extraction conditions for PCRs targeting tropical pathogens from stool are therefore desirable.

## 5. The Choice of the Assay and Validation-Associated Issues

Numerous in-house and commercial PCR assays have been developed for the detection of bacterial gastroenteric pathogens from stool samples since the first introduction of this technique for travellers returning from the tropics in 1994 [2]. With a strong focus on *C. difficile* [66,67,68,69,70,71,72,73,74,75,76,77] and *Campylobacter* spp. [78,79,80,81], which play important roles as causative agents also in resource-rich Western industrialized countries, the portfolio of published in-house and commercial PCR assays for potential use in the tropics also comprises enteroinvasive bacteria like *S. enterica* and *Shigella* spp., in part in large panels [82,83,84,85,86,87,88,89,90,91,92], but also diarrheagenic *E. coli* [93,94].

Although the high number of published papers on PCR targeting bacterial gastroenteric pathogens may mediate the impression of indifference regarding the assay of choice, there are indeed some aspects to be considered. The German microbiological and infectiological quality standards (MIQ) recommend against the diagnostic use of traditional gel-based or SybrGreen-based PCRs with melting curve analysis, because cross-reaction cannot be reliably excluded based on just two DNA-binding oligonucleotides [95]. Accordingly, a confirmation step either by restriction endonuclease treatment or by probe binding or by sequencing of the amplicon is demanded to ensure sufficiently high specificity [95]. As restriction endonuclease treatment is, however, quite laborious and sequencing still comparably expensive, probe binding as part of real-time PCR is the most frequently chosen approach to comply with such quality standards. Next to this, each PCR run has to be accompanied by positive controls, negative controls as well as nucleic acid extraction and inhibition controls. In case of desired quantification, control samples with defined copy numbers of the target genes have to be included as well [95].

However, such technical quality control is still insufficient for diagnostic purposes. In silico validation of the applied oligonucleotides and in vitro validation of the whole assay are also mandatory prior to diagnostic use [95,96]. Unfortunately, no internationally accepted validation standards for in vitro diagnostic tests exist, although precise knowledge of the diagnostic accuracy of applied in vitro assays is crucial for both individual clinical and study outcomes, so it has been argued that they should be similarly rigorously evaluated as therapeutic drugs [97].

The test validation scheme as suggested by Rabenau and colleagues for viral pathogens [98] is internationally widely acknowledged. However, it demands the availability of a reliable reference standard.

It might be argued that diagnostic stool culture could be applied as a reference standard. However, diagnostic yield of stool culture is known to be low [99,100,101] and PCR can also detect DNA from avital bacteria, making culture a reference standard of questionable sensitivity for PCR targeting DNA of bacterial causes of gastroenteritis. The issue is likely to be aggravated in case of studies in the tropics due to the challenges regarding storage and transport conditions, as detailed above in the pre-analytics chapter.

Without a reliable reference standard, however, indirect methods for diagnostic accuracy estimation have to be considered as summarized elsewhere [102]. Such indirect approaches comprise Bayesian estimations requiring information on the expected prevalence [103], comparisons of assays including sample populations with unknown but most likely different prevalence values under the assumption of stable test performance with both populations, as well as the use of reference tests with imperfect but known diagnostic accuracy [104], and last but not least, latent class analysis (LCA) [105]. All those mathematical approaches have certain prerequisites which have to be considered as more or less guaranteed for their application [102]. In spite of their undeniable disadvantages [102], those strategies are nevertheless options in order to come as close as possible to the aim of measuring “absolute truth”, an aim which is aspired to but never reached by any diagnostic method [96].

Within the European Union, steps are being taken regarding the standardization of diagnostic test validation by the imminent enforcement of the Regulation (EU) 2017/746 [106,107]. In short, the enforcement will lead to a need for the application of certified tests unless additional benefit of in-house approaches is proven. Although it is basically a good idea to hold the test-producing industry accountable for high-quality validation of their diagnostic products, it is likely that the high validation standards as demanded by the Regulation (EU) 2017/746 will lead to a vanishing of commercial PCR kits for rarely assessed parameters, because the marketing of such test kits may not be cost efficient. This is potentially bad news for rare and neglected tropical pathogens, for which diagnosis will then be restricted to few reference centers instead of becoming broadly available.

Even in case of well-validated test assays with good sensitivity and specificity values of 99% or more, the predictive values depend on pretest probability according to Bayes’ theorem [102]. Accordingly, in case of low prevalence with resulting low pretest probability, the positive predictive value will necessarily be low in spite of good but not perfect specificity of the applied assay. This has to be considered in case of PCR-based screening in low-prevalence settings or if PCR-based diagnosis of very rare tropical infections is intended.

Further, when interpreting diagnostic PCR results, one has to keep in mind that the target structure of each PCR assay is just a defined nucleic acid sequence, not the pathogen as a whole. The pathogen is the meta-structure, for which this sequence acts as a sensitive surrogate parameter [102]. Thereby, sensitivity depends on the stability of the target sequence, i.e., the reliability of its occurrence in unaltered homology in all target pathogens in all geographic regions of interest. More than this, the surrogate status of the target sequence can also affect a PCR assay’s specificity if the assay is used under circumstances different from its validation setting. In particular, in stool samples, myriads of microorganisms exist, whose composition varies considerably depending on factors such as geography, ethnicity and subsistence [108]. So, if PCR targeting a certain nucleic acid sequence is specific for a defined pathogen in human gut microbiomes at the validation site, it is not completely excluded that a very similar sequence might occur in non-target microorganisms in the stool of individuals in another geographic region, potentially leading to cross-reactions and associated false positive results. Similarly, altered variants or reduced copy numbers of the target sequence might occur in target pathogens in certain geographic regions, which might limit sensitivity. Accordingly, it is recommendable to perform regional validations as well before a PCR assay targeting gastroenteric pathogens is transferred from one region of the world to another. This particularly applies to remote tropical settings, for which the pre-existing knowledge on the composition of the gut microbiomes of the local population is scarce [108]. No in silico validation of PCR oligonucleotides can be better than the quality of available sequence data and the reliability of each in vitro validation will necessarily depend on the representativeness of the available sample material.

If reliable sequence databases can be considered as granted, sequencing of the amplicon is a good option in order to confirm the specificity of a positive PCR result [95]. Due to the associated effort and costs, however, this option is rarely chosen.

In case of surveillance or study settings, imperfect test accuracy of PCR assays can be compensated by accuracy-adjusted prevalence estimators [109,110] or LCA-based prevalence estimation [105], as long as test accuracy is known from evaluation studies. Such approaches, however, are helpful on a population level only, and cannot decide the correctness of a defined PCR result for an individual patient. Considering the lacking generally accepted validation standards for PCR assays and the abovementioned limitations, more experience with PCR-based diagnosis for bacterial gastroenteric pathogens in the tropics will be highly desirable to help physicians better estimate the clinical impact of such PCR results. Academic assessment of harmonization of PCR targeting gastrointestinal bacterial pathogens in tropical conditions will thus remain a domain of implementation science.

## 6. Post-Analytics–the “Infection or Colonization” Decision

Apart from the abovementioned issues of uncertain test specificity under circumstances different from the validation setting and low positive predictive value in case of low pretest probability [102], there is yet another aspect to consider when interpreting the results of PCRs targeting gastroenteric pathogens in the tropics.

When PCR from stool of patients with diarrhea is performed in resource-rich Western industrialized countries like Germany [83] or Switzerland [111], even after travelling in the tropics under hygienically controlled circumstances [111,112], only individual pathogens are usually detected that are then causally attributed to a coexisting gastrointestinal disease. Under tropical conditions, however, such etiological attribution is not as easy, because DNA of typical gastrointestinal pathogens such as *S. enterica*, *Campylobacter* spp., diarrheagenic *E. coli* [7,13,14,30] and even *Shigella* spp. [19] or enteropathogenic protozoa [7,13,14,18,19,30,113] can be detected in asymptomatic individuals in the tropics as well. Adaptation processes and semi-immunity are considered to account for such phenomena in case of detection of facultatively pathogenic causative agents in asymptomatic individuals [114]. Even in symptomatic patients, however, attribution of etiological relevance of individual pathogens is challenging if more than one pathogen is detected, as is frequently the case in resource-limited tropical settings with poor hygiene conditions [7,11,13].

Next to asymptomatic colonization [114,115,116], persistence of DNA of successfully treated and already cleared infections [24] can also account for positive PCR results without association to symptomatic gastroenteritis. DNA clearance from stool, either by excretion or nucleases, is a stochastic process and contradicting reports of DNA persistence duration after cleared infections between a few days and several weeks within the complex stool matrix exist [113,117].

In particular, the discrimination of etiologically relevant infection from harmless colonization is a considerable challenge for PCR applied with stool samples [103]. Some authors have argued that lower cycle threshold (Ct) values of real-time PCR, corresponding to higher pathogen loads, might be used for the discrimination of colonization and infection [118,119,120]. In particular for *Shigella* spp., there have been repeated reports indicating higher likeliness of etiological relevance in case of recorded low Ct values [118,120]. However, other studies failed to reproduce such associations [19,30,116] and clear or at least gradual cut-offs for respective discrimination attempts are missing so far. Likely reasons for the lacking success in establishing reliable associations between Ct values and clinic relevance of pathogen detection might include semi-immunity [114] but also varying diagnostic accuracy of applied PCR assays, either due to the design of the assays or due to altered distribution of target sequences as well as potentially cross-reacting non-target sequences in various geographic regions. Accordingly, such associations need to be assessed and stratified by both PCR assay and geographic region, demanding a considerable workload and ongoing standardization of the applied assays to make their results comparable.

Discrimination of colonization from infection in case of positive PCR results will most likely remain a challenge in settings where semi-immunity due to repeated pathogen exposition [114] associated with poor hygiene conditions is prevalent. As long as approaches in order to drastically increase the hygiene levels in resource-limited tropical countries are lacking, however, frequent spread of fecal–orally transmitted pathogens will go on triggering semi-immunity [114]. Further diagnostic studies addressing this issue of etiological relevance, e.g., by including also data on local gut microbiome compositions [108] or parameters indicating inflammatory reactions as also suggested for other infectious diseases [121,122,123,124,125,126], are required.

## 7. Discussion

The review was performed to focus on some yet to be resolved challenges for the reliable application of diagnostic PCR for bacterial agents causing gastroenteritis with focus on the situation in the tropics, where infectious diarrhoea is a particular issue of concern as repeatedly shown for both the local population [7,13] and travellers [127,128]. As detailed above, a number of open research questions which should be addressed in the near future have been identified as summarized in Table 1.

If well-equipped laboratory infrastructure is scarce, automated cartridge-based PCR approaches provide an easy-to-implement solution for PCR in resource-poor tropical settings, as successfully proven by the rollout of GenXpert-(Sunnyvale, CA, USA-)based tuberculosis screening in sub-Saharan Africa [129,130,131,132]. Such assays are considerably easier to implement compared to traditional multiplex real-time PCR assays [133] and also available for gastroenteric pathogens [29]. However, open or hidden costs of automated PCR approaches, which have already been described for automated PCR applied for tuberculosis screening [134,135], make the broader implementation of such technologies in the resource-poor tropics for an infectious disease as frequent as infectious diarrhoea, apart from study settings, challenging.

The choice of the most appropriate PCR assays for the tropics remains a challenge, because validation studies specifically performed in the tropics are still scarce. Although even broad confidence intervals regarding the expected test accuracy due to a paucity of validation data can be included in the calculation of test accuracy-adjusted prevalence on a population-based level, such considerations [109,110] are of little practical help for individual diagnostic approaches and resulting therapeutic decisions.

At least specificity-associated problems with the interpretation of PCR results can in part be compensated by sequencing if a sufficient amount of amplicon DNA is available. Some authors even argue that unbiased sequencing approaches directly from primary sample materials may provide useful hints in the future [136]; however, polymicrobial contamination makes the interpretation of the results of such approaches challenging if assessments from primary non-sterile sample materials such as, e.g., gut biopsies are performed [137]. Additionally, although modern molecular diagnostic assays are quite robust, suboptimal sample material is always a challenge for the diagnostic threshold even in case of modern PCR platforms [138].

PCR-based diagnosis of causative agents of bacterial gastroenteritis in other compartments such as, e.g., in blood in case of bacteremic shedding, remains challenging due to low pathogen concentration in the bloodstream. As detailed above, assessment by PCR with or without subsequent sequencing is possible after blood culture incubation as well [50,139], but requires laborious sample preparation to avoid PCR inhibition. Highly repetitive long sequence targets like retrotransposons, which allow the PCR-based detection of single cells of *Schistosoma* spp. in peripheral blood [140], are usually scarcely available in bacterial genomes.

The broad variety of potential causative agents of infectious gastroenteritis remains another challenge. This includes emerging new pathogens, as recently demonstrated by the causal association of SARS-CoV-2 detection in a stool sample of a child with diarrhoea in Iran [141].

In a more abstract epistemiological sense, medical diagnosis is always a variant of the art of coming close to an unknown truth [96]. As described above, PCR-based diagnosis of bacterial gastroenteritis in the tropics is quite a challenging variant of this art and various scientific questions still need to be addressed in this field in the future.

## 8. Conclusions

In summary, PCR for the diagnosis of gastrointestinal pathogens is a widely applied approach, whose potential for a diagnostic routine application under tropical conditions requires additional evaluation. Further investigations are particularly needed to standardize the pre-analytic, analytic and post-analytic steps with special emphasis on the climatic, infrastructural, epidemiological and socioeconomic situation in the tropics. With adequate academic support by the disciplines of implementation science, however, it will most likely be possible to define optimum conditions for beneficial use of this technique under tropical conditions as well.

## Figures and Tables

**Table 1:** Challenges for PCR targeting bacterial pathogens causing gastroenteritis with particular focus on the situation in the resource-limited tropics.

Stage of the Diagnostic Process	Challenge
Pre-analytic stage	Lacking definition of minimum standards regarding sample acquisition, storage and transport (time) for diagnostic analyses in the tropics.
Diagnostic stage–nucleic acid extraction	Lacking agreement on nucleic acid extraction standards which allow an optimum detection of all types of pathogens which may be responsible for gastroenteritis in the tropics.
Diagnostic stage–PCR	Lacking internationally accepted validation standards to ensure comparable quality of applied diagnostic assays.
Post-analytic stage–result interpretation	Lacking cut-offs for a reliable discrimination of infection and mere colonization in areas where semi-immunity due to repeated exposition associated with poor hygiene conditions has to be expected.

## Data Availability

Not applicable.
